# Determination of patient-specific internal gross tumor volumes for lung cancer using four-dimensional computed tomography

**DOI:** 10.1186/1748-717X-4-4

**Published:** 2009-01-27

**Authors:** Muthuveni Ezhil, Sastry Vedam, Peter Balter, Bum Choi, Dragan Mirkovic, George Starkschall, Joe Y Chang

**Affiliations:** 1 Department of Radiation Oncology, The University of Texas M. D. Anderson Cancer Center, Houston, USA; 2 Department of Radiation Physics, The University of Texas M. D. Anderson Cancer Center, Houston, USA

## Abstract

**Background:**

To determine the optimal approach to delineating patient-specific internal gross target volumes (IGTV) from four-dimensional (4-D) computed tomography (CT) image data sets used in the planning of radiation treatment for lung cancers.

**Methods:**

We analyzed 4D-CT image data sets of 27 consecutive patients with non-small-cell lung cancer (stage I: 17, stage III: 10). The IGTV, defined to be the envelope of respiratory motion of the gross tumor volume in each 4D-CT data set was delineated manually using four techniques: (*1*) combining the gross tumor volume (GTV) contours from ten respiratory phases (IGTV_AllPhases_); (*2*) combining the GTV contours from two extreme respiratory phases (0% and 50%) (IGTV_2Phases_); (*3*) defining the GTV contour using the maximum intensity projection (MIP) (IGTV_MIP_); and (*4*) defining the GTV contour using the MIP with modification based on visual verification of contours in individual respiratory phase (IGTV_MIP-Modified_). Using the IGTV_AllPhases _as the optimum IGTV, we compared volumes, matching indices, and extent of target missing using the IGTVs based on the other three approaches.

**Results:**

The IGTV_MIP _and IGTV_2Phases _were significantly smaller than the IGTV_AllPhases _(*p *< 0.006 for stage I and *p *< 0.002 for stage III). However, the values of the IGTV_MIP-Modified _were close to those determined from IGTV_AllPhases _(*p *= 0.08). IGTV_MIP-Modified _also matched the best with IGTV_AllPhases_.

**Conclusion:**

IGTV_MIP _and IGTV_2Phases _underestimate IGTVs. IGTV_MIP-Modified _is recommended to improve IGTV delineation in lung cancer.

## Background

Lung cancer remains the leading cause of cancer-related mortality. Conventional photon radiotherapy for lung cancer is associated with about 50% local tumor control [1]. Missing the target as a result of tumor motion has been considered one of the main reasons for local failure [2]. Researchers have reported that ~40% of lung tumors move > 5 mm and that 10–12% move > 1 cm [3,4]. Several strategies have recently been developed to address the issue of tumor motion and improve local control [2]. For example, the development of image-guided radiotherapy (IGRT) has allowed for more accurate tumor targeting, so it is rapidly replacing conventional radiotherapy for lung cancer [2]. In order to account for tumor motion, the International Commission on Radiation Units and Measurements (ICRU) report 62 introduced the concept of an internal target volume (ITV), defined as the clinical target volume (CTV) plus an additional margin to account for geometric uncertainties due to internal variations in tumor position, size, and shape. Using current imaging techniques, the CTV cannot be visualized. Consequently, generation of the ITV requires delineation of the gross tumor volume (GTV) on each of the phases that constitute the four-dimensional (4-D) computed tomography (CT) image data set, followed by expansion of each GTV to account for microscopic disease. The ITV is then determined to be the envelope of motion of the CTV. In order to make the determination of the ITV more efficient, we have proposed the concept of the internal gross tumor volume (IGTV), which explicitly accounts for internal variations in tumor position, size, and shape but can be derived directly from imaging studies [2]. The ITV is then determined to be the IGTV plus a margin that accounts for microscopic disease.

Traditionally, the margin necessary to account for internal motion of tumors in the thorax has been determined using an isotropic expansion determined by population-based estimates of respiratory motion. However, because breathing characteristics vary greatly among individual patients, such population-based estimates may overestimate or underestimate the margin needed for a given patient. Moreover, respiratory-induced tumor motion is known not to be anisotropic; typical tumor paths are those of elongated and possible curved ellipses. The advent of the multislice helical CT scanner combined with the establishment of temporal correlation between respiratory motion and the CT acquisition process have allowed tumor size, shape, and position to be observed at multiple times during a patient's respiratory cycle [5,6]. The resultant CT data set, called the 4-D CT or respiration-correlated CT data set, provides patient-specific information about tumor position, shape, and size at different phases of the respiratory cycle.

Although using 4-D CT data provides a reliable estimate of the extent of tumor motion due to respiration in three dimensions, its clinical implementation poses some challenges. Ideally, the IGTV should be determined by contouring the GTV on each of the ten phase image sets. The combination of these individual three dimensional (3-D) volumes into a single 3-D volume represents the IGTV, which accounts for respiratory motion. However, contouring the tumor volume on ten different data sets for each patient increases the workload compared with contouring in only one dataset. In these instances, post-processing tools, such as the maximum intensity projection (MIP), have been shown to improve radiotherapy planning efficiency [7]. The MIP of a 4D-CT data set reduces the multiple 3-D CT data available from a 4-D CT data set into a single 3-D CT data set, where each voxel in the MIP represents the maximum intensity encountered by corresponding voxels in all individual 3-D phase image sets of the 4-D CT data set. The IGTV is then determined based on the GTV delineation on the single 3-D CT data set. Alternatively, some cancer centers have used breath-hold spiral CT imaging to acquire images at the two extremes of the respiratory cycle [2,7]; contouring the GTV at these extremes (the end-expiration and the end-inspiration phases) and then combining these two 3-D volumes yields the IGTV. A limited number of studies have analyzed the accuracy of the MIP and two-phase IGTV delineation techniques relative to full ten-phase method for determining IGTV [8-11].

The aim of this study, therefore, was to evaluate the accuracy of 4-D CT MIP-based IGTV delineation and two-phase-based IGTV delineation compared to ten-phase IGTV delineation as a reference. We also examined the accuracy of the MIP-based IGTV delineation after applying a modification through visual verification of GTV coverage in individual respiratory phases.

## Methods

### Data acquisition

As a retrospective review of radiation treatment planning, this study was included under an Institutional Review Board-approved retrospective chart review protocol. We studied 27 consecutive patients with non-small-lung cancer (NSCLC) who underwent 4-D CT simulation for treatment planning and received definitive radiotherapy at our institution between 2005 and 2006. Of these 27 patients, 17 had stage I disease and received stereotactic body radiotherapy (SBRT), and 10 had stage III disease and received intensity-modulated radiotherapy (IMRT). 4-D CT image data sets each consisting of 10 respiratory phases, were acquired on a multislice CT scanner (Discovery ST, GE Medical Systems, Madison, WI) by sorting CT images based on the phase of an external respiratory monitor (Real-time Position Management System; Varian Medical Systems, Inc., Palo Alto, CA) [12]. MIPs of the 4D-CT data sets were then generated from the individual phase images as described elsewhere [5,6].

### Patient-specific IGTV determination

**Figure 1:**
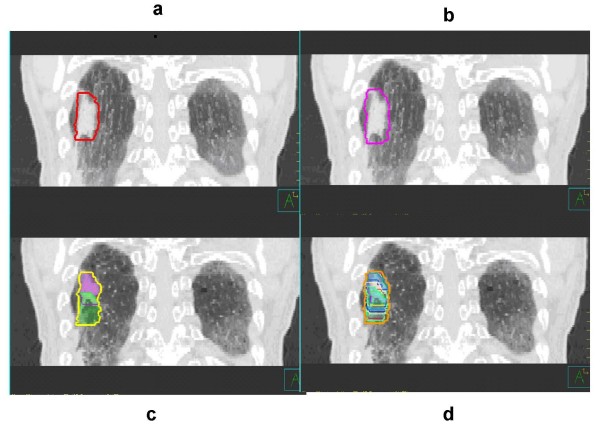
**Delineation of IGTV for stage I lung tumors based on (a) IGTV_MIP_, (b) IGTV_MIP-Modified_, (c) IGTV_2Phases_, and (d) IGTV_AllPhases _of a 4-D CT data set**. MIP-based contours, as shown in panels (a) and (b), are as they appear on the MIP data set. Phase-based contours, as shown in panels (c) and (d), are registered to the peak exhalation phase of the 4-D CT data set.

**Figure 2:**
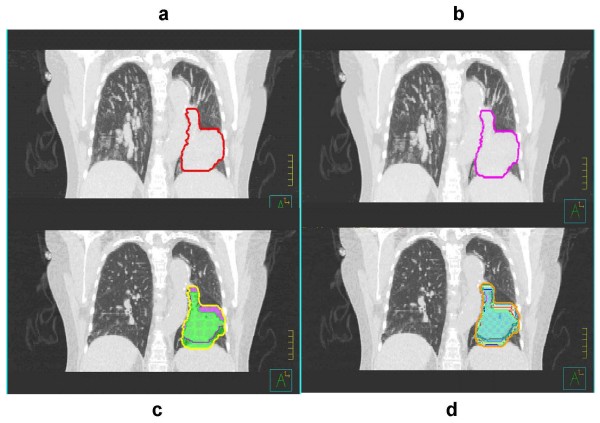
**Delineation of IGTV for stage III lung tumors based on (a) IGTV_MIP_, (b) IGTV_MIP-Modified_, (c) IGTV_2Phases_, and (d) IGTV_AllPhases _of a 4-D CT data set**. MIP-based contours, as shown in panels (a) and (b), are as they appear on the MIP data set. Phase-based contours, as shown in panels (c) and (d), are registered to the peak exhalation phase of the 4D-CT data set.

We determined patient-specific IGTVs using the demonstrable extent of tumor motion shown in the 4-D CT images. We used four approaches to determine these IGTVs: (*1*) contouring the GTV on each of the ten respiratory phases of the 4D-CT data set and combining these GTVs to produce IGTV_AllPhases_; (*2*) contouring the GTV on the MIP of the 4-D CT data set to produce IGTV_MIP_; (*3*) contouring the GTV on the extreme respiratory phases (0% phase = peak inhalation, 50% phase = peak exhalation) and combining these GTVs to produce IGTV_2Phases_; and (*4*) contouring the GTV on the MIP of the 4-D CT data set and then modifying these contours using visual verification of coverage in each phase of the 4-D CT data set to produce IGTV_MIP-Modified_. Visual verification of coverage in each phase was achieved by overlaying the MIP based GTV contour onto each phase of the 4-D CT data set. Thus, each of these 3D volumes (IGTV_AllPhases_, IGTV_MIP_, IGTV_2Phases_, and IGTV_MIP-Modified_) represented the demonstrable respiratory tumor motion volumes, or IGTVs. Figures 1 and 2 show the results obtained using these different approaches in the determination of IGTV for cases of stage I and stage III disease, respectively. For consistency in contouring, all GTV contours in each respiratory phase of the 4-D CT and MIP data sets were drawn by a single radiation oncologist (ME) and verified by another radiation oncologist (JYC). We used a lung window on the CT data set to contour the primary tumor and a mediastinum window to contour any involved lymph nodes. Diagnostic CT of chest with intravenous contrast and PET/CT were used to guide our involved lymph nodes contouring as described by our previous publication (2). A total of 324 GTVs were delineated with 12 GTVs delineated for each patient (GTV in each of 10 respiratory phases, IGTV_MIP_, and IGTV_MIP-Modified_). For stage III disease, involved hilar or mediastinal lymph nodes were contoured and analyzed independently.

### Data analysis

We evaluated the IGTVs determined using each of the three contouring approaches against an all phases IGTV determined by contouring all ten respiratory phases of the 4-D CT data set (IGTV_AllPhases_). Specifically, we compared the following metrics for each 3D volume: matching index, total GTV volume and under or over-estimated volume.

### Matching index calculation

The matching index (MI) of any two 3D volumes A and B is defined as the ratio of the intersection of A with B to the union of A and B, that is,

$$MI=\frac{A\cap B}{A\cup B}.$$

As can be deduced from this equation, the maximum value of the MI is 1 if the two volumes are identical, and the minimum value is 0 if the volumes are completely non-overlapping.

### Volume difference calculation

**Figure 3:**
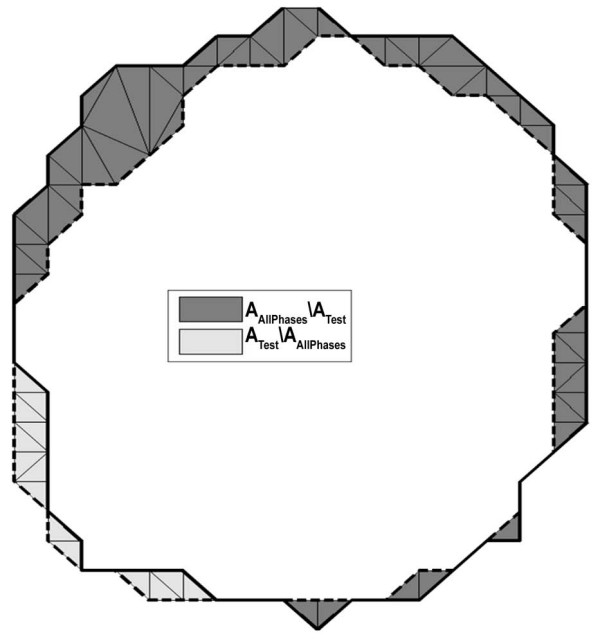
**Computation of the underestimation area (dark gray) and the overestimation area (light gray) of the test area (area inside the dashed line) compared with reference area (area inside the solid line)**. The areas were computed using the Delaunay triangulation which is shown in the regions of interest.

While the matching index is a good measure of how well the shape of any two volumes match each other, it cannot discriminate between overestimation and underestimation. To gain better insight into any over/underestimation of the IGTV, we computed the differences in IGTV between the all phases volume (IGTV_AllPhases_) and the three test volumes (IGTV_MIP_, IGTV_2Phases_, and IGTV_MIP-Modified_). For each pair of volumes, we computed the underestimation and overestimation volumes (*V*_*Under *_and *V*_*Over*_) using the following equations:

$$\begin{array}{c} V_{Under}=V_{AllPhases}\backslash V_{Test}\\ V_{Over}=V_{Test}\backslash V_{AllPhases}, \end{array}$$

where *V*_*AllPhases *_is the volume in ten respiratory phases, *V*_*test *_is the test volume, and "\" denotes the set difference. The underestimation and overestimation volumes were computed as integrals over the *z *coordinate of the corresponding transverse areas as follows:

$$\begin{array}{l} V_{Under}=\int A_{AllPhases}(z)\backslash A_{Test}(z)dz,\\ V_{Over}=\int A_{Test}(z)\backslash A_{AllPhases}(z)dz, \end{array}$$

where *A*_*AllPhases *_is the area in ten respiratory phases and *A*_*Test *_is the test area. The underestimation area (*A*_*Under*_) and the overestimation area (*A*_*Over*_) defined as

$$\begin{array}{l} A_{Under}=A_{AllPhases}(z)\backslash A_{Test}(z),\\ A_{Over}=A_{Test}(z)\backslash A_{AllPhases}(z), \end{array}$$

were computed for each axial level by performing the Delaunay triangulation for the union of the all phases and test contour points and computing the areas as a sum of the corresponding triangular areas (see Figure 3). Given a set of data points in the plane, the Delaunay triangulation is a set of triangles such that no data points are contained in any triangle's circumscribed circle. Delaunay triangulations maximize the minimum angle of all the triangles in the triangulation and they tend to avoid skinny (or close-to-degenerate) triangles. We used the Delaunay triangulation implemented in a high-level graphical analysis and programming package, MATLAB (The Mathworks, Inc.: ), which is based on the Quickhull algorithm [13].

### Statistical analysis

To estimate any statistically significant differences between the IGTVs determined using each test volume (IGTV_MIP_, IGTV_2Phases_, and IGTV_MIP-Modified_) and the IGTV determined using the all phases volume (IGTV_AllPhases_), we used a paired sample *t*-test in each case to determine *p*, with *p *< 0.05 considered significant. All statistical analyses were performed using the SPSS software package (v.10; SPSS Inc., Chicago, IL).

## Results

Table 1 shows the superior-inferior (SI) motion and the IGTVs based on the test and all phases volumes for the stage I lung tumors. SI motion ranged from 0 cm to 2.17 cm, with almost half (8/17) of the tumors exhibiting SI motion > 1 cm. To study the influence of magnitude of SI motion on the accuracy of IGTV delineation, we grouped the 17 patients into two groups: those with tumor motion > 1.00 cm and those with tumor motion ≤1.00 cm. In general, we found that, regardless of the magnitude of SI motion, the IGTV_MIP _and IGTV_2Phases _(mean ± SD: 14.14 ± 14.89 cm^3 ^and 13.93 ± 15.69 cm^3^, respectively) were consistently smaller than the IGTV_AllPhases _(mean ± SD: 16.60 ± 17.05 cm^3^), whereas the IGTV_MIP-Modified _(mean ± SD: 16.33 ± 16.67 cm^3^) were similar to the reference IGTV. A paired sample *t*-test revealed that the IGTV_MIP _and IGTV_2Phases _differed significantly from the IGTV_AllPhases_(*p *< 0.001), while the IGTV_MIP-Modified _did not differ significantly from the reference IGTV (*p *= 0.08).

**Table 1:** SI motion and IGTVs based on the test volumes (IGTV_MIP_, IGTV_2Phases_, and IGTV_MIP-Modified_) and the reference volume (IGTV_AllPhases_) for stage I tumors

Patient No	SI Motion (cm)	IGTV_MIP_ (cm^3^)	IGTV_MIP-Modified_ (cm^3^)	IGTV_AllPhases_ (cm^3^)	IGTV_2Phases_ (cm^3^)
1	1.06	5.82	7.98	8.32	6.56

2	1.37	8.53	10.51	9.98	7.49

3	1.70	12.88	15.85	16.54	13.23

4	1.08	4.92	5.49	5.44	3.72

5	0.15	1.64	1.79	1.80	1.46

6	2.17	17.64	22.16	23.39	18.98

7	1.27	23.08	26.06	26.28	21.77

8	0.54	12.45	15.73	15.76	12.82

9	0.18	21.80	24.50	24.96	20.92

10	0.00	60.04	66.90	67.75	63.67

11	0.41	2.46	2.80	2.85	2.27

12	1.77	32.90	37.65	39.39	33.14

13	0.14	2.08	2.27	2.23	1.84

14	0.10	1.53	1.90	1.93	1.74

15	1.62	18.59	21.26	21.69	16.66

16	0.66	10.70	11.31	10.60	7.77

17	0.09	3.35	3.45	3.33	2.70

Table 2 shows the MI values for each of the three test IGTVs. As shown, the IGTV_MIP-Modified _(mean ± SD: 0.90 ± 0.02) most closely matched the IGTV_AllPhases_, with IGTV_2Phases _(mean ± SD: 0.81 ± 0.06) and IGTV_MIP _(mean ± SD: 0.80 ± 0.05) following. There were no significant differences between IGTV_2Phases _and IGTV_MIP _(*p *= 0.728), but the differences in MI between IGTV_MIP _and IGTV_MIP-Modified _and those between IGTV_2Phases _IGTV_MIP-Modified _were significant (*p *< 0.001, respectively)

**Table 2:** Matching index values for each IGTV based on IGTV_MIP_, IGTV_2Phases_, and IGTV_MIP-Modified _relative to the reference IGTV_AllPhases _in stage I disease

Patient No	Location (Adjacent)	IGTV_MIP_	IGTV_2Phases_	IGTV_MIP-Modified_
1	Diaphragm	0.69	0.79	0.88

2	Diaphragm	0.79	0.75	0.88

3	Diaphragm	0.77	0.80	0.92

4	Chest wall	0.82	0.68	0.90

5	Lung parenchyma	0.83	0.81	0.88

6	Chest wall	0.72	0.80	0.89

7	Lung parenchyma	0.84	0.83	0.91

8	Mediastinum	0.74	0.81	0.92

9	Chest wall	0.84	0.84	0.91

10	Chest wall	0.87	0.94	0.95

11	Lung parenchyma	0.79	0.80	0.90

12	Chest wall	0.83	0.84	0.93

13	Lung parenchyma	0.82	0.83	0.90

14	Lung parenchyma	0.75	0.90	0.91

15	Diaphragm	0.79	0.77	0.89

16	Lung parenchyma	0.85	0.73	0.88

17	Lung parenchyma	0.88	0.81	0.91

We performed a comparative analysis of the MI values of the two patient groups (patients with SI motion ≤1 cm and those with SI motion > 1 cm) with stage I disease. There was no strong correlation between the MI and the magnitude of SI motion, although the MI of IGTV_2Phases _in some patients with SI motion ≤1 cm was lower than the general trend in patients with SI motion > 1 cm. Although the magnitude of SI motion did not significantly impact the accuracy of the IGTV contouring approaches, we found that the location of the primary tumor impacted IGTV contouring accuracy (Table 2). For example, we found that tumors located near the diaphragm (cases 1, 2, 3, and 15), mediastinum (case 8), and chest wall (cases 4, 6, 9, 10, and 12) appeared to have worse MI values than tumors located in the peripheral lung parenchyma (cases 5, 7, 11, 13, 14, 16, and 17) although it didn't reach statistical significance.

Table 3 shows the SI motion and the IGTVs based on the test and all phases volumes for the 10 stage III lung tumors. As shown, the majority of these tumors (9/10) exhibited SI motion < 1 cm, so it was not meaningful to group these patients according to the 1-cm-SI motion threshold.

**Table 3:** SI motion and IGTVs based on the test volumes (IGTV_MIP_, IGTV_2Phases_, and IGTV_MIP-Modified_) and the reference volume (IGTV_AllPhases_) for stage III tumors

Patient No	SI Motion (cm)	IGTV_MIP_ (cm^3^)	IGTV_MIP-Modified_ (cm^3^)	IGTV_AllPhases_ (cm^3^)	IGTV_2Phases_ (cm^3^)
1	0.09	64.91	77.33	79.94	74.42

2	0.12	202.85	228.55	238.40	216.29

3	0.21	135.59	146.62	151.40	138.72

4	0.18	221.50	230.85	233.74	222.22

5	0.62	23.87	30.59	29.98	23.33

6	0.11	446.14	450.31	458.21	439.86

7	0.96	242.38	265.47	268.58	244.76

8	0.14	347.06	368.97	373.61	351.46

9	0.18	36.69	39.40	36.87	34.03

10	1.77	211.70	221.96	228.89	203.01

As with stage I lung tumors, we found that, regardless of the magnitude of SI motion, the IGTV_MIP _and IGTV_2Phases _(mean ± SD: 193.27 ± 135.09 cm^3 ^and 194.81 ± 133.86 cm^3^, respectively) were consistently smaller than the IGTV_AllPhases _(mean ± SD: 209.96 ± 139.95 cm^3^), whereas the IGTV_MIP-Modified_(mean ± SD: 206.00 ± 137.34 cm^3^) was similar to the all phases IGTV. A paired sample *t*-test revealed that the IGTV_MIP _and IGTV_2Phases _differed significantly from the IGTV_AllPhases _(*p *< 0.001), while the IGTV_MIP-Modified _differed less (*p *= 0.01).

Table 4 shows the MI values for each IGTV based on the test volumes and on the all phases volume for patients with stage III disease. In general, we found that the GTV_MIP-Modified_-based IGTV (mean ± SD: 0.93 ± 0.20) matched the GTV_AllPhases_-based IGTV the closest, followed by the IGTVs based on GTV_2Phases _(mean ± SD: 0.91 ± 0.05) and GTV_MIP _(mean ± SD: 0.86± 0.07). There was a significant difference between GTV_2Phases_-based and GTV_MIP_-based IGTVs (*p *= 0.05) and between GTV_MIP_-based and GTV_MIP-Modified_-based IGTVs (*p *= 0.03).

**Table 4:** Matching index values for each IGTV based on IGTV_MIP_, IGTV_2Phases_, and IGTV_MIP-Modified _relative to the reference IGTV_AllPhases _in stage III disease

Patient No	GTV_MIP_	GTV_2Phases_	GTV_MIP-Modified_
1	0.76	0.93	0.92

2	0.83	0.91	0.92

3	0.88	0.92	0.94

4	0.92	0.95	0.95

5	0.74	0.78	0.90

6	0.95	0.96	0.95

7	0.87	0.91	0.94

8	0.90	0.94	0.94

9	0.87	0.92	0.91

10	0.86	0.89	0.90

The volumetric underestimation and overestimation between the all phases volume and the test volumes for patients with stage I and III disease are shown in Table 5. For stage I disease, the maximum volumetric underestimations for IGTV_MIP_, IGTV_2Phases_, and IGTV_MIP-Modified _compared to IGTV_AllPhases _were 30.86%, 21.2%, and 8.53%, respectively. For stage III disease, the maximum volumetric underestimations for IGTV_MIP_, IGTV_2Phases_, and IGTV_MIP-Modified _compared to IGTV_AllPhases _were 23.85%, 22.25%, and 6.66%, respectively. The average volumetric underestimation was 17.3% for IGTV_MIP_, 19.3% for IGTV_2Phases_, and 5.3% for IGTV_MIP-Modified _in stage I tumors and 12.1% for IGTV_MIP_, 8.9% for IGTV_2Phases_, and 4.2% for IGTV_MIP-Modified _in stage III tumors. In sum, we found that the volumetric underestimation for IGTV_MIP-Modified _was consistently lower than the underestimation for IGTVs based on the other test volumes. We also observed that the volumetric underestimation percentages in stage III disease were lower than those in stage I disease. However, because GTVs are by definition larger in stage III than in stage I disease, the absolute volume underestimation was generally higher in stage III disease. Volumetric overestimation occurred in both stage I and stage III disease for both IGTV_MIP _and IGTV_MIP-Modified_. Overestimation for IGTV_MIP-Modified _was slightly higher than that for IGTV_MIP_, but both percentages were lower than 5.0% for the average volume overestimation and 10.10% for the maximum volume overestimation. Because IGTV_2Phases _is a subset of IGTV_AllPhases_, the volumetric overestimation for IGTV_2Phases _compared to the reference IGTV was always equal to zero.

**Table 5:** Summary of the volumetric percentage underestimation and overestimation for each IGTV based on IGTV_MIP_, IGTV_2Phases_, and IGTV_MIP-Modified _relative to the reference IGTV_AllPhases_.

Underestimation (%)		IGTV_MIP_	IGTV_2Phases_	IGTV_MIP-Modified_
Stage I patients				
				
	Avg. ± SD	17.33 ± 6.56	19.32 ± 5.93	5.36 ± 1.71
				
	Range	6.32–30.86	6.03–31.76	2.30–8.53
				
				

Stage III patients				
				
	Avg. ± SD	12.11 ± 6.23	8.95 ± 5.15	4.21 ± 1.66
				
	Range	4.04–23.85	4.01–22.25	1.20–6.66
				
				

Overestimation (%)		IGTV_MIP_	IGTV_2Phases_	IGTV_MIP-Modified_

Stage I patients				
				
	Avg. ± SD	3.23 ± 2.35	0	4.80 ± 2.39
				
	Range	0.34–8.64	0	1.30–10.07
				
				

Stage III patients				
				
	Avg. ± SD	2.36 ± 1.79	0	3.21 ± 2.22
				
	Range	1.06–6.92	0	1.43–8.09

Average ± standard deviation and range are reported for stage I and stage III tumors.

**Figure 4:**
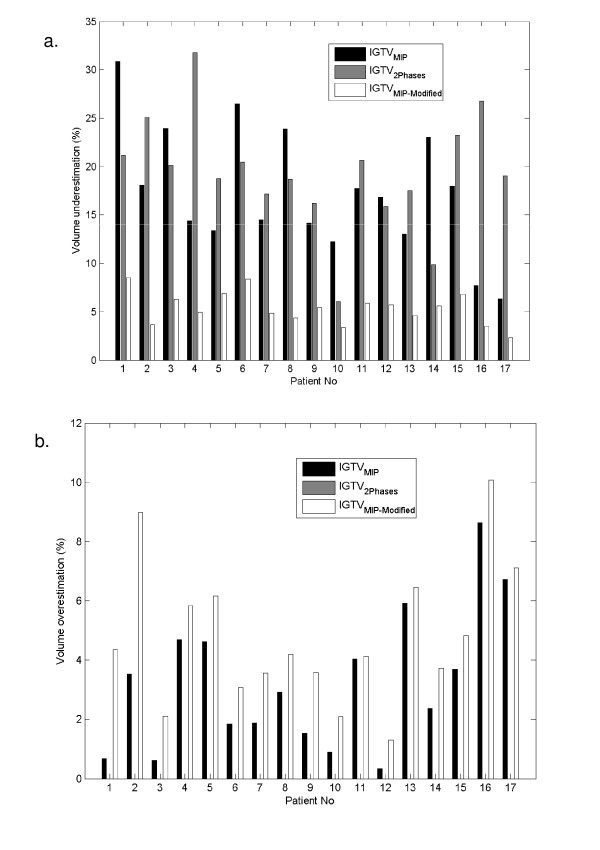
**Stage I tumors**. (a) Volumetric underestimation for each IGTV based on IGTV_MIP_, IGTV_2Phases_, and IGTV_MIP-Modified _relative to the reference IGTV_AllPhases_. (b) Volumetric overestimation for each IGTV based on IGTV_MIP_, IGTV_2Phases_, and IGTV_MIP-Modified _relative to the IGTV_AllPhases_. (Note: IGTV_2Phases _is a subset of IGTV_AllPhases_, hence the volumetric overestimation for IGTV_2Phases _is always equal to zero.)

**Figure 5:**
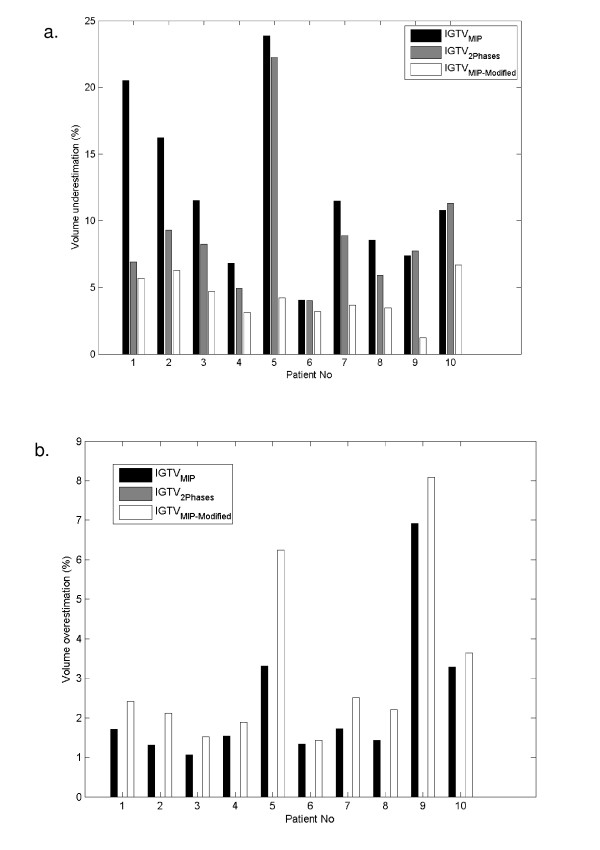
**Stage III tumors**. (a) Volumetric underestimation for each IGTV based on IGTV_MIP_, IGTV_2Phases_, and IGTV_MIP-Modified _relative to the IGTV_AllPhases_. (b) Volumetric overestimation for each IGTV based on IGTV_MIP_, IGTV_2Phases_, and IGTV_MIP-Modified _relative to the IGTV_AllPhases_. (Note: IGTV_2Phases _is a subset of IGTV_AllPhases_, hence the volumetric overestimation for IGTV_2Phases _is always equal to zero.)

Figure 4 illustrates the proportional volumetric underestimations (Fig. 4a) and overestimations (Fig. 4b) in the 17 individual patients with stage I disease. We found that volumetric underestimation was > 10% using either IGTV_MIP _or IGTV_2Phases _in 15 patients, but in no patients when IGTV_MIP-Modified _was used. Volumetric underestimation > 20% occurred in 5 patients using the IGTV_MIP _and in 7 patients using the IGTV_2Phases_. Of the 5 patients in whom volumetric underestimation was > 20% using IGTV_MIP_, 2 had lesions near or attached to the diaphragm, 1 had a lesion near or attached to the chest wall, and another had a lesion near or attached to the mediastinum. Figure 5 illustrates the volumetric underestimations (Fig. 5a) and overestimations (Fig. 5b) in the 10 patients with stage III disease. We found that volumetric underestimation was > 5% in 9 patients using IGTV_MIP_, 8 patients using IGTV_2Phases_, and 2 patients using IGTV_MIP-Modified_. Volumetric underestimation > 10% occurred in 6 patients using IGTV_MIP_, 1 patient using IGTV_2Phases_, but no patients using IGTV_MIP-Modified_. In general, we found that the lowest volumetric underestimation was achieved consistently using the modified MIP approach to delineate the IGTV.

To analyze the accuracy of these contouring approaches in involved lymph nodes, we conducted the second analysis of involved lymph nodes in above stage III disease. Our data showed that IGTV_MIP-Modified _volume of lymph nodes (mean ± SD: 32.95 ± 40.86 cm^3^) matched most closely with IGTV_AllPhases _volumes of lymph nodes (mean ± SD: 34.26 ± 42.56 cm^3^, p = 0.24), while IGTV_2Phases _and IGTV_MIP _lymph node volumes (mean ± SD: 29.15 ± 38.14 and 25.63 ± 34.55 cm^3 ^respectively) differed significantly with IGTV_AllPhases _lymph node volume (p = 0.04 and 0.05 respectively, volume underestimation in all cases). In addition, the match index of lymph node IGTV_MIP-Modified _was not significantly different from IGTV_2Phases _(p = 0.14) but was significantly different from IGTV_MIP _values (p = 0.001 for both cases). IGTV_MIP-Modified _and IGTV_2Phases _matched better with IGTV_AllPhases _(match index mean ± SD: 0.81 ± 0.08, range: 0.75–0.91 for IGTV_MIP-Modified_, and 0.77 ± 0.08, range: 0.65 to 0.88 for IGTV_2Phases_) compared with IGTV_MIP _(mean ± SD: 0.62 ± 0.11; Range: 0.46 to 0.76).

## Discussion

Real-time tumor motion tracking provides most comprehensive data for respiratory tumor motion management. However, it is a challenging technique to implement in the clinical setting and more research is needed to make its clinical implementation more practical [14]. Although both MIP-based and two-phase-based approaches have been shown to more accurately delineate the GTV than conventional 3D CT-based planning, their accuracy has not been compared with that of ten-phase contouring approach particularly in stage III disease. Jin *et al*, in a phantom study, examined the feasibility of a method to determine ITV based on motion information obtained from select phases of a respiratory cycle [15]. They reported that adequate estimation of IGTV could in general be achieved by combining motion information from the extremes of motion in most cases and in some cases by the addition of motion information from an intermediate phase. Underberg *et al*. [8] reported that MIP-based contouring could provide reliable margins for determining the IGTV for stage I lung tumors treated with SBRT. However, their method did not include visual verification of the MIP-defined GTV contour through each individual phase of the 4D CT (IGTV_MIP-Modified_). Bradley *et al*. [9] compared helical-, MIP-, and average-intensity (AI)-based 4-D CT imaging to find the optimal approach for determining the patient-specific IGTV for SBRT for stage I lung cancer. They found that the MIP-defined GTV was significantly larger than the helical-defined and average CT-defined GTVs. However, in their study, Bradley *et al*. did not compare the GTV based on GTV_MIP _with that based on GTV_AllPhases_, the optimal reference volume. Bradley *et al*. [9] did not discuss their results in the context of tumor location in their study. In another study, Cai *et al*. [10] determined the IGTVs for six lung tumors using a simulation method based on dynamic magnetic resonance imaging (dMRI) and MIPs. They found that MIP-based IGTVs were smaller than dMRI-based IGTVs. They concluded that because of the low temporal resolution and retrospective re-sorting, 4-D CT might not accurately depict the excursion of a moving tumor. Recent data by Rietzel *et al *also support our observation that tumor delineation on the MIP with subsequent visual verification of contours over all individual phases of the 4D CT yielded the best estimate of IGTV. However, there the performance of this approach in the delineation of involved lymph nodes was not separately addressed [11]. In daily clinical practice, tumor contouring in stage III disease is more challenging than in stage I disease because of the larger tumor volume, more complicated tumor shape, involvement of critical structures, and potential involvement of multiple lymph nodes in which tissue density is similar to that of the tumor. In addition, although the two-phase-based approach has been used to delineate IGTVs in the clinical setting, there is scant data on the accuracy of such two-phase-based IGTVs in either stage I or stage III disease [16]. Our study showed that both MIP-based and two-phase-based IGTVs underestimate the 10-phase-based IGTV in both stage I and III disease including involved lymph nodes, which can potentially result in marginal under-dosing, and that the IGTV_MIP-Modified _consistently had the lowest percentages of volumetric underestimation, which indicates that the IGTV_MIP-Modified _approach is the most accurate in delineating the IGTV.

For the MIP-based approach, several potential sources of uncertainty/error exist: (*1*) the MIP image may not fully display mobile structures if the adjacent structures have similar (or higher) densities, which is the case for lesions located near the mediastinum, diaphragm, liver, or chest wall; and (*2*) the physician may misinterpret the MIP images because of tumor border smearing. (*3*) The tumor spicula can not be visualized on the MIP projections due to smearing of the tumor edge. Indeed, our data show that the MI was poor and volumetric underestimation was high using the MIP-based approach to delineate IGTVs in most of lesions near the mediastinum, diaphragm, liver, and chest wall. Of these lesions, those closer to the diaphragm and liver had the lowest MI values, which could have been due to the significant motion of the diaphragm and liver and the MIP image's inability to record differences between the lesion and the diaphragm and liver. We are currently developing software that excludes diaphragm and liver images in some breathing phases using cine CT images so that better tumor MIP images will be preserved (data to be published). We should note that MIP images do not reflect the densities of tumors, lungs, and other normal tissues accurately enough for dose calculation in treatment planning [17]. Thus, a free-breathing CT image set, a 4-D scan of a single respiratory phase, or an average CT image set extracted from a 4-D CT data set should be used for treatment planning and dose calculation. This would be especially important in proton therapy, which is more sensitive to tumor motion and changes in tissue density. In a previous study on 4-D CT in proton therapy planning, we found that a MIP density override for tumor contouring in an average CT data set was the optimal approach [18].

For the two-phase-based approach, tumor deformation between the two extreme phases of breathing and the curved motion pathway during each breathing cycle may introduce uncertainty. In most cases, however, we found that the MI of the two-phase-based IGTV was slightly higher than that of MIP-based IGTV, which indicates that most tumors moved in a generally straightforward SI direction and that tumor deformation during breathing was minimal. Particularly in stage III disease, we found that the volumetric underestimation was generally lower for the two-phase-based IGTV than for the MIP-based IGTV. Therefore, if 4-D CT based IGTV_MIP-Modified_is not available, the two-phase-based IGTV is a reasonable alternative approach to take tumor motion into consideration although it is not optimal one.

In clinical setting, it is common to prescribe the dose to PTV which takes additionally clinical target volume (CTV) and set-up uncertainty into consideration. The volume-underestimation will be reduced if PTV was used to compare above mentioned four approaches. We evaluated the effect of this underestimation on the PTV in a case with maximal underestimation of the IGTV in stage I disease. IGTV was expanded by 1.6 cm (0.8 cm for CTV, 0.3 cm to account for variability in the determination of motion extent and 0.5 cm for image guided patient setup). Analysis of volumetric underestimation of the PTV was carried out in the same manner as described for IGTV. Our results showed that the volume underestimation reduced from 30.86%, 21.2%,8.53% in IGTV to 13.3%, 5.18% and 3.36% in PTV for IGTV_MIP_, IGTV_2Phases_, IGTV_MIP-Modified _respectively. In general, this improvement is more dramatic in the lesions with the smaller size such as stage I disease. However, when ablative dose is attempted in clinical setting but sparing critical structures is concerning such as SBRT in stage I disease, we would accept compromised coverage for PTV but not for IGTV. Therefore, IGTV delineation accuracy is still crucial clinically.

As with other such comparative studies mentioned above, inter or intra observer variability in the delineation of the GTV was not considered. The uncertainties introduced as a result of the above could however be thought to be different from those analyzed in this study, thereby requiring a separate analysis that is beyond the scope of the current report.

## Conclusion

We found that the MIP-based and two-phase-based approaches to IGTV delineation significantly underestimated the IGTV in patients with stage I and stage III NSCLC. Due to the limitations of each approach, a significant amount of the tumor volume could be missed in individual patient so precautions should be taken when these techniques are used to treat patients. We also found that the IGTV_MIP-Modified _approach, which requires visual verification of tumor coverage after each phase of the breathing cycle, improved IGTV delineation in both cases.

## Abbreviations

GTV: gross tumor volume; IGTV: internal gross tumor volume; CTV: Clinical target volume; PTV: Planning target volume; IGTV_AllPhases_: the gross tumor volume (GTV) contours from ten respiratory phases; IGTV_2Phases_: the GTV contours from two extreme respiratory phases (0% and 50%); IGTV_MIP_: the GTV contour using the maximum intensity projection (MIP); IGTV_MIP-Modified_: the GTV contour using the MIP with modification based on visual verification of contours in individual respiratory phase.

## Competing interests

The authors declare that they have no competing interests.

## Authors' contributions

ME, SV, and JYC designed/conducted analysis and wrote the manuscript. JYC was responsible for manuscript revision and submission. PB, BC and GS were involved in 4-D CT simulation and treatment designed. DM was involved in data analysis.

## Authors' information

Dr. Chang is a recipient of the Research Scholar Award from the Radiological Society of North America and a Development Award from The University of Texas M. D. Anderson Cancer Center NIH Lung Cancer SPORE (P50 CA70907).
